# How do patients pass through stroke services? Identifying stroke care pathways using national audit data

**DOI:** 10.1177/0269215520907654

**Published:** 2020-03-06

**Authors:** Matthew Gittins, David G Lugo-Palacios, Lizz Paley, Benjamin Bray, Audrey Bowen, Andy Vail, Brenda Gannon, Sarah Tyson

**Affiliations:** 1 Centre for Biostatistics, The University of Manchester, Manchester, UK; 2 Manchester Academy for Health Sciences, Manchester, UK; 3 Manchester Centre for Health Economics, The University of Manchester, Manchester, UK; 4 Sentinel Stroke National Audit Programme, King’s College London, London, UK; 5 Division of Neuroscience & Experimental Psychology, The University of Manchester, Manchester, UK; 6 School of Economics, The University of Queensland, Brisbane, QLD, Australia; 7 Division of Nursing, Midwifery & Social Work, The University of Manchester, Manchester, UK

**Keywords:** Stroke, physiotherapy, occupational therapy, speech and language therapy, clinical psychology

## Abstract

**Objective:**

To map and describe how patients pass through stroke services.

**Methods:**

Data from 94,905 stroke patients (July 2013–July 2015) who were still inpatients 72 hours after hospital admission were extracted from a national stroke register and were used to identify the routes patients took through hospital and community stroke services. We sought to categorize these routes through iterative consultations with clinical experts and to describe patient characteristics, therapy provision, outcomes and costs within each category.

**Results:**

We identified 874 routes defined by the type of admitting stroke team and subsequent transfer history. We consolidated these into nine distinct routes and further summarized these into three overlapping ‘pathways’ that accounted for 99% of the patients. These were direct discharge (44%), community rehabilitation (47%) and inpatient transfer (19%) with 12% of the patients receiving both inpatient transfer and community rehabilitation. Patients with the mildest and most severe strokes were more likely to follow the direct discharge pathway. Those perceived to need most therapy were more likely to follow the inpatient transfer pathway. Costs were lowest and mortality was highest for patients on the direct discharge pathway. Outcomes were best for patients on the community rehabilitation pathway and costs were highest where patients underwent inpatient transfers.

**Conclusion:**

Three overarching stroke care pathways were identified which differ according to patient characteristics, therapy needs and outcomes. This pathway mapping provides a benchmark to develop and plan clinical services, and for future research.

## Introduction

Since the late 1990s, when the Stroke Unit Trialists established that specialist stroke unit care improved outcomes,^1^ there has been a sea-change in stroke services through the adoption of evidence-based care. Specialist stroke units are now the default way of organizing stroke care. When first introduced, they focussed on providing specialist stroke rehabilitation and acute care was provided in general medical wards; however, the advent of acute stroke care as a clinical speciality led to a shift towards acute, and then hyper-acute stroke unit care^2^ Recently, a ‘hub and spoke’ model in which patients are taken to a large, central specialist unit (rather than the nearest hospital) for hyper-acute stroke before repatriation to the local stroke service for ongoing care has demonstrated greater clinical effectiveness and cost-effectiveness and is being implemented in large metropolitan areas.^3^ Specialist stroke services after discharge from hospital, in the form of early supported discharge and community rehabilitation teams, have also proved more clinical- and cost-effective^4–6^ than hospital services without postdischarge rehabilitation, and have been widely implemented: 80% of the stroke services in the United Kingdom now have access to an early supported discharge service and 75% have access to a community stroke rehabilitation service.^7^

Although these different types of specialist stroke units/teams are effective, recommended in clinical guidelines and have been widely implemented,^8^ they each address a specific stage of stroke care. Stroke services, however, need to cover all stages of care, from admission to long-term support for ‘life after stroke’. There are many ways of configuring these overarching stroke services. They may consist of any combination of hyper-acute, acute, inpatient rehabilitation, early supported discharge and/or community rehabilitation either as stand-alone or as combined units or teams. The way these overarching services are configured may affect (positively or negatively) stroke outcomes beyond that reported for an individual type of stroke unit/teams. Our aim in this article was to explore how stroke patients passed through stroke services in terms of the types of stroke units/teams they were treated by from admission to hospital to final discharge from specialist stroke services. Specifically, we wished to categorize routes to facilitate service planning and research for patients with different therapy needs, describing outcomes and costs in each category. This was the first step of work to investigate the effectiveness of different therapy provision in practice.

## Methods

Data from the Sentinel Stroke National Audit Programme (SSNAP) were used. Since 2013, SSNAP has operated as a national register to audit stroke care in England, Wales and Northern Ireland, collecting data on over 95% of all stroke events. As such, it offers a unique opportunity to investigate the stroke care delivered in the real world. The programme has three components: clinical patient-level audit recorded continuously,^9^ and acute organizational,^10^ and postacute organizational^11^ audits that are recorded every two years. The clinical component is a longitudinal register that collects information about patients’ characteristics, clinical status and the care they receive for all patients during inpatient stroke care and is reported on a voluntary response basis during post-inpatient care. Data include demographics, details of the stroke, treatment and health outcomes. Full details on SSNAP can be found elsewhere.^9^

We adopted the classification of stroke teams used in the SSNAP organizational audit in 2014^10^ to define the types of stroke team:

- Routinely admitting team: stroke teams which *regularly directly admit* stroke patients for acute and/or hyper-acute stroke care;
- Non-routinely admitting team: teams which *do not generally admit* stroke patients directly but provide acute care after patients transferred from their place of initial treatment, which is typically from a hyper-acute stroke team;
- Non-admitting inpatient team: teams which *do not admit* stroke patients but provide inpatient stroke rehabilitation;
- Early supported discharge team: multidisciplinary teams which coordinate early discharge from hospital and provide short-term community rehabilitation typically for patients with mild-moderate stroke;^12^
- Community rehabilitation team: multidisciplinary teams which provide community rehabilitation for stroke patients with any level of severity. The timescale over which treatment is provided varies but is generally longer than an early supported discharge team;
- Integrated community rehabilitation team: a team that provided both early supported discharge and community rehabilitation.

To distinguish between routinely admitting teams providing acute (and hyper-acute) care only, or ‘combined’ teams which provided acute care and rehabilitation, the routinely admitting teams were further classified according to their median length of stay. SSNAP defines acute stroke care as lasting up to seven days,^10^ so teams with a median length of stay of less than seven days were defined as providing hyper-acute and acute care and referred to as an *acute stroke team*. Teams with a median length of stay of seven days or more were referred to as a *combined stroke team*. The community-based services refer to both stroke-specific and generic community-based teams that reported their data to SSNAP.

The *route* a patient takes through stroke services was defined by the combination of types of stroke team they were treated by during inpatient and community stroke care: in other words, from first hospital admission, any transfers between inpatient and/or community-based teams, to final discharge from specialist stroke care. The order of the teams was also noted. Patients following routes with similar characteristics which could be used to define and evaluate different ways of organizing stroke care were grouped. This was done such that high-frequency common routes were retained and low-frequency routes were combined with others with common characteristics, leading to a mutually exclusive and comprehensive categorization. Decisions regarding how the routes were combined were taken in consultation with multidisciplinary consultation group with clinical and academic expertise in all relevant professions and stages of stroke care. The route categories thus defined were further consolidated into clinically meaningful *pathways*.

The patients within each pathway were characterized in terms of their patient demographics, stroke characteristics, therapy received and health outcomes using standard descriptive statistics. Stroke severity was categorized according to the score on the National Institute of Health Stroke Scale (NIHSS) on admission as follows: <5 = mild stroke, 5–14 = moderate stroke, 15–20 = severe stroke, and >20 = very severe stroke.

The large sample sizes and strong confounding factors present in this observational data set precluded simplistic, unifactorial analysis of differences between the pathways. Such analyses would be subject to high risk of bias, exacerbated by the large sample size resulting in apparent statistical significance.

We calculated the cost of each pathway using the National Health Service Reference Costs 2014–2015^13^ to compute the costs associated with inpatient stroke care in each pathway. The costs associated with community-based rehabilitation were computed using the SSNAP cost and cost-effectiveness analysis.^13,14^ The reference cost collection is the single national collection of service costs within the National Health Service. This collection reports the average unit cost to the National Health Service provider for each currency or spell of health care in England in a given financial year.^15^ They include direct, indirect and overhead costs and emphasize the cost of delivering the service. They do not provide information on the variation of costs between patients receiving the same health care activity, nor the location of the service or the funding streams used to recover these costs.^15,16^ The costs of inpatient stroke care were estimated for each patient using the average cost of non-elective stays, the length of stay within each stroke team and the pathway followed. When a patient was transferred to a new hospital, or discharged and readmitted, this is considered a new spell of stroke care and its respective average cost was added to the total average cost for that patient. Transfers between different stroke teams within the same hospital were considered part of the same spell of care. The costs associated with community-based stroke care were calculated from the type and amount of therapy and the average cost per patient applied.^14^ Information on the cost per visit (for physiotherapy, occupational therapy and speech therapy) or per hour (for psychology) was taken from the SSNAP cost-effectiveness analysis assuming patients had one visit of each therapy on the days they received treatment.^14^ The cost for psychological therapy was computed per hour as this was the information available from the analysis.^14^ Inpatient and community care costs were computed for each patient using the data contained in Supplementary Data and averaged per pathway.

The analyses were undertaken on a data set extracted from the SSNAP database. This involved all patients admitted to hospital with a confirmed diagnosis of stroke between July 2013 and July 2015 (the most up-to-date data available at the start of the project) in England, Wales and Northern Ireland. As the project involved secondary analysis of anonymized, routinely collected clinical data, ethical approval was not required. This article is part of a larger project to investigate the real-world delivery of stroke therapy (physical, occupational and speech therapy plus psychology), and thus, we excluded patients who died, were discharged or were transferred for palliative care within the first three days of the admission as they were likely to have little need for stroke therapy (beyond assessment). Patients who had a high degree of missing data on the NIHSS^17^ (which measured stroke impairments and severity on admission) were also excluded as the incompleteness of these data prevented us from characterizing the patients’ stroke. Thus, patients who had a score for level of consciousness (which is mandatory to complete on SSNAP) but all other items were unreported were excluded. A few patients were readmitted after they had been discharged from specialist stroke care. The data from their initial period of care were retained, but those from their readmission were excluded in order to reflect only their planned care.

## Results

**Figure 1.**
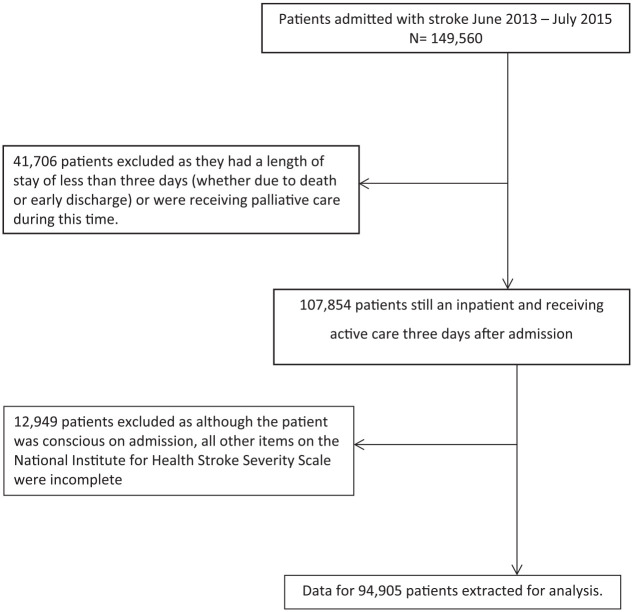
Flow chart depicting selection criteria.

The data set extracted from SSNAP involved 94,905 patients once the exclusion criteria had been applied (Figure 1), 314 of whom were readmitted. A total of 874 distinct routes were identified, of which 500 included five patients or fewer while the 20 most populated routes involved 70% of the patients (Online Appendix 1). Iterative categorization led to the definition of nine routes (Table 1). These were the eight possible combinations in which initial admission was to an acute or combined stroke team, whether inpatient transfers occurred and whether community-based rehabilitation was provided, plus a small group (1.2%) who were not initially admitted to either an acute or a combined stroke team.

**Table 1.** Definition of routes and clinical pathways.

Pathway	Route	Admitting team	Transfer(s) to further inpatient care	Community rehabilitation after inpatient discharge	No. of patients (%)
Direct discharge	R1	Acute	No	No	21,647 (22.8)
	R2	Combined	No	No	19,779 (20.8)
Community rehabilitation	R3	Acute	No	Yes	17,308 (18.2)
	R4	Combined	No	Yes	17,250 (18.2)
Inpatient transfer	R5	Acute	Yes	Yes	8410 (8.9)
	R6	Combined	Yes	Yes	1810 (1.9)
	R7	Acute	Yes	No	6479 (6.8)
	R8	Combined	Yes	No	1067 (1.1)
Other	R9	Other	Yes or no	Yes or no	1155 (1.2)

Acute: routinely admitting team with median length of first stay < seven days; combined: routinely admitting team with median length of first stay > seven days; community-based rehabilitation: community rehabilitation team or early supported discharge team; other: not routinely admitting team.

Routes 5 and 6 in the shaded region appear in both the community rehabilitation and the inpatient transfer pathways.

Four pathways were defined (Table 1):

- *Direct discharge pathway* (Routes 1 and 2): discharged from specialist stroke care (usually home or to residential care) directly from their admitting stroke team;
- *Community rehabilitation pathway* (Routes 3–6): transferred to community-based rehabilitation on discharge from hospital;
- *Inpatient transfer pathway* (Routes 5–8): transferred from the admitting team to further inpatient team(s) for rehabilitation;
- *Other pathway* (Route 9): initial admission to somewhere other than a routinely admitting team.

Ninety-nine percent of the patients were admitted to a routinely admitting team (57% to acute stroke team and 42% to combined team). Forty-four percent of the cohort (*n* = 41,426) followed the simplest, direct discharge pathway with similar numbers admitted to an acute stroke team (Route 1, 22.8% of the cohort) and a combined team (Route 2, 20.8% of the cohort).

The community rehabilitation pathway involved 44,778 patients (47.2% of the whole cohort) and four routes. Patients who were discharged to community rehabilitation directly from their admitting team formed Route 3 (if discharged from an acute team, *n* = 17,308 (18.2%)) and Route 4 (if discharged from a combined team, *n* = 17,250 (18.2%)). Patients who were transferred to another inpatient team before discharge with community rehabilitation formed Route 5 (*n* = 8410, 8.9%) and Route 6 (*n* = 1810, 1.9%).

Nineteen percent of the cohort (*n* = 17,766) were treated by more than one inpatient stroke team and followed the inpatient transfer pathway, 80% of whom (*n* = 14,213) were transferred only once before discharge. The maximum number of stroke teams that a patient was treated by was 8 (*n* = 1). This inpatient transfer pathway involved four routes in which patients were discharged from stroke care with (Route 5 if originally admitted to an acute team, and Route 6 if originally admitted to a combined team) or without community rehabilitation (Routes 7 and 8, respectively). Route 7 involved 6479 (6.8% of the whole cohort), and Route 8 involved 1067 (1.1% of the whole cohort). Typically, the transfers were to stroke teams which focused on rehabilitation, such as non-routinely admitting team or a specialist stroke rehabilitation team (or non-admitting inpatient unit). Patients in this pathway had, overall, the most severe strokes and the demand for therapy was the high.

Patients who followed the direct discharge pathway, Routes 1 and 2, had similar demographics to each other (Table 2). On average, they were a few years older than those on the community rehabilitation pathway (Routes 3–6) and similar to those on Routes 7 and 8. The sex distribution was similar across all routes and pathways. The possible differences between ethnic and social deprivation groups were difficult to interpret in the light of missing data.

**Table 2.** Demographic characteristics by route and pathway.

	Direct discharge		Community rehabilitation		Inpatient transfer				Other	Total
	R1	R2	R3	R4	R5	R6	R7	R8	R9	
Age (years)										
Mean (*SD*)	78 (13.1)	77 (13.1)	74 (13.1)	74 (12.9)	73 (13.4)	74 (12.7)	78 (13.1)	77 (12.7)	75 (14.6)	76 (13.2)
Sex										
Female	11,867 (24)	10,658 (22)	8478 (17)	8449 (17)	4033 (8)	885 (2)	3635 (7)	583 (1)	611 (1)	49,199
Male	9780 (21)	9121 (20)	8830 (19)	8801 (19)	4377 (10)	925 (2)	2844 (6)	484 (1)	544 (1)	45,706
Ethnicity										
White	19,563 (23)	18,694 (22)	15,517 (18)	16,098 (19)	6379 (8)	1698 (2)	4956 (6)	1005 (1)	88 (3)	84,816
Asian	424 (16)	340 (13)	474 (18)	379 (14)	570 (21)	15 (1)	367 (14)	12 (0.4)	89 (7)	2669
Black	200 (15)	130 (10)	177 (13)	94 (7)	408 (30)	8 (1)	255 (19)	4 (0.3)	16 (5)	1365
Mixed	44 (15)	48 (16)	49 (17)	50 (17)	45 (15)	4 (1)	35 (12)	3 (1)	34 (1)	294
Unknown	1211 (26)	487 (11)	951 (21)	554 (12)	677 (15)	76 (2)	592 (13)	38 (1)	22 (2)	4620
Other	205 (18)	80 (7)	140 (12)	75 (7)	331 (29)	9 (1)	274 (24)	5 (0.4)	906 (1)	1141
Social deprivation (quartiles: least (1) to most (4) deprived)										
1	4519 (21)	4199 (19)	4179 (19)	4466 (20)	2185 (10)	342 (2)	1498 (7)	121 (1)	413 (1.9)	21,922
2	5478 (21)	3950 (18)	4289 (19)	3757 (17)	2295 (10)	397 (2)	1784 (8)	146 (1)	281 (1.3)	22,377
3	5402 (24)	4515 (20)	4267 (19)	4055 (18)	2022 (9)	423 (2)	1452 (6)	176 (1)	223 (1.0)	22,535
4	4805 (23)	4517 (22)	3929 (19)	3745 (18)	1592 (8)	339 (2)	1222 (6)	151 (1)	194 (0.9)	20,494
Missing	1443 (19)	2598 (34)	644 (9)	1227 (16)	316 (4)	309 (4)	523 (7)	473 (6)	44 (0.6)	7577
Total	21,647 (23)	19,779 (21)	17,308 (18)	17,250 (18)	8410 (9)	1810 (2)	6479 (7)	1067 (1)	1155 (1)	94,905

The shaded area denotes the routes which are common to two pathways.

The median stroke severity was 6 (on NIHSS scale) for the direct discharge Pathway (Routes 1 and 2); 5 for those on the community rehabilitation Pathway after discharge from the admitting team (Routes 3 and 4); 7 for community rehabilitation after an inpatient transfer (Routes 5 and 6) and higher for those who had an inpatient transfer before discharge without community rehabilitation (Routes 7 and 8) (Table 3). Those with mild/very mild, severe/very severe strokes were over-represented in the direct discharge Pathway. Patients with severe/very severe stroke were under-represented in Routes 3 and 4. Those on the direct discharge pathway had the lowest perceived need for therapy. Those on inpatient transfer pathway had the greatest need for therapy.

**Table 3.** Clinical characteristics by route and clinical pathway.

	Direct discharge pathway		Community rehabilitation pathway		Inpatient transfer pathway				Other	Total^a^
	R1	R2	R3	R4	R5	R6	R7	R8	R9	
Stroke severity (NIHSS)										
Median (IQR)	6 (3–15)	6 (2–14)	5 (2–9)	5 (2–9)	7 (4–13)	7 (4–14)	9 (5–17)	8 (4–15)	4 (2–9)	6 (3–12)
Mild (<5)	7499 (21)	7699 (21)	7895 (22)	8003 (22)	2626 (7)	501 (1)	1425 (4)	262 (1)	466 (1)	36,376
Moderate (5–14)	7978 (21)	6767 (18)	7300 (20)	6908 (18)	3922 (11)	866 (2)	2859 (8)	466 (1)	461 (1)	37,527
Severe (15–20)	2833 (27)	2450 (23)	1229 (12)	1307 (12)	1041 (10)	258 (3)	1080 (3)	190 (2)	117 (1)	10,505
Very severe (>20)	3337 (32)	2863 (27)	884 (8)	1032 (10)	821 (8)	185 (2)	1115 (11)	149 (1)	111 (1)	10,497
Prestroke										
mRS ⩽ 2	15,657 (21)	14,653 (20)	15,042 (20)	14,783 (20)	7080 (9)	1620 (2)	4548 (6)	870 (1)	848 (1)	75,101
Haemorrhage	19,119 (23)	17,580 (21)	15,615 (19)	15,482 (18)	7127 (9)	1553 (2)	5537 (7)	922 (1)	993 (1)	83,928
Needing therapy										
Physiotherapy	19,195 (18)	17,549 (17)	16,680 (16)	16,643 (16)	17,012 (16)	3571 (3)	12,523 (12)	1959 (2)	1162 (1)	106,294
Occupational therapy	17,655 (17)	16,073 (15)	16,611 (16)	16,528 (16)	16,893 (16)	3453 (3)	11,850 (12)	1809 (2)	1129 (1)	102,001
Speech therapy	11,888 (18)	10,633 (16)	9437 (14)	9742 (14)	11,854 (18)	2286 (3)	9433 (14)	1283 (2)	758 (1)	67,314
Psychology	661 (9)	848 (11)	891 (12)	1242 (16)	2091 (27)	559 (7)	1059 (14)	206 (3)	140 (2)	7647

Routes 5 and 6 in the shaded region appear in both the community rehabilitation and the inpatient transfer pathways.

a Based on 115,247 admissions of 94,905 patients.

NIHSS, National Institute of Health Stroke Scale; IQR, interquartile range, mRS, modified Rankin Scale.

Patients on the direct discharge pathway, whether Route 1 or Route 2, least frequently required therapy and received the least therapy per day of inpatient stay (Table 4). Those on the community rehabilitation pathway received relatively large amounts of therapy as inpatients as well as receiving therapy after hospital discharge. The amount of community therapy received was similar in all the routes in the community rehabilitation pathway and was less than that received while an inpatient. Patients following Routes 7 and 8 received less inpatient therapy than those on Routes 5 and 6. Patients in the inpatient transfer pathway had the greatest need for therapy and received the most therapy.

**Table 4.** Outcomes by route and clinical pathway.

Pathway	Route	Inpatient therapy (min per day)^a^				Community therapy (min per day)^a^				Length of inpatient stay	Independence (mRS ⩽ 2) at final discharge	Mortality
		PT	OT	SLT	Psych	PT	OT	SLT	Psych			
DD	R1	12.2 (9)	11.2 (10)	6.9 (6)	3.0 (4)	/	/	/	/	8.7 (5,25)	8219 (38%)	5853 (27%)
	R2	12.4 (10)	11.3 (9)	7.3 (6)	1.9 (3)	/	/	/	/	10.2 (5,31)	7990 (40%)	5393 (27%)
CR	R3	16.3 (10)	17.8 (12)	9.1 (8)	3.4 (4)	12.6 (13)	11.9 (14)	8.7 (11)	2.1 (3)	8.6 (5,19)	9974 (58%)	93 (1%)
	R4	16.1 (11)	16.5 (11)	9.1 (8)	2.4 (3)	11.2 (12)	10.0 (13)	6.8 (8)	2.7 (4)	13.5 (7,31)	9383 (54%)	123 (1%)
IT	R5	21.0 (10)	20.3 (11)	11.9 (9)	5.0 (6)	11.9 (12)	10.4 (14)	7.3 (12)	1.8 (3)	30.9 (15,56)	3063 (36%)	112 (0.1%)
	R6	19.9 (9)	16.4 (9)	9.1 (7)	2.6 (3)	12.1 (10)	9.0 (9)	7.2 (8)	2.2 (2)	54.2 (33,79)	624 (34%)	13 (1%)
	R7	17.2 (9)	14.9 (10)	10.1 (8)	4.8 (6)	/	/	/	/	38.2 (16,67)	1297 (20%)	1543 (24%)
	R8	14.9 (9)	11.7 (9)	7.1 (7)	1.9 (2)	/	/	/	/	57.0 (30,97)	262 (25%)	174 (3%)
Other	R9	13.4 (10)	14.1 (11)	7.9 (7)	3.1 (4)	11.0 (12)	9.3 (12)	5.8 (8)	1.3 (2)	17.9 (9,39)	419 (36%)	200 (17%)

a Figures are median (interquartile range).

mRS, modified Rankin Scale; PT, physical therapy; OT, occupational therapy; SLT, speech-language therapy; DD, direct discharge pathway; CR, community rehabilitation pathway; IT, inpatient transfer pathway.

Length of inpatient stay was always shorter if admitted to an acute team than to a combined stroke team, regardless of subsequent inpatient transfers or community rehabilitation. The difference in medians ranged from 1.5 days (Route 1 vs. Route 2) to over three weeks (Route 5 vs. Route 6). Unsurprisingly, routes on the inpatient transfer pathway had the longest median lengths of inpatient stay. Independence (modified Rankin Scale ⩽ 2) at final discharge was highest in Routes 3 and 4 (community rehabilitation without inpatient transfer) and lowest in Routes 7 and 8 (inpatient transfer without community rehabilitation). Mortality was lowest (<1%) in the community rehabilitation pathway and highest (27%) on the direct discharge pathway.

Overall, patients on the community rehabilitation had the mildest strokes (on average) and were most frequently independent before their stroke. This was particularly noticeable for patients in Routes 3 and 4 (discharged to community rehabilitation from the admitting team). Patients following Routes 5 and 6 (community rehabilitation after an inpatient transfer) were more severely affected. Despite having relatively mild strokes, patients in the community rehabilitation pathway had the highest demand for therapy of all the pathways. Nearly all patients required physical therapy and occupational therapy, while the demand for speech therapy and psychology was 55%–56% and 5%–15%, respectively.

Average total costs were lowest in Routes 1–4. High mortality in the direct discharge pathway obviously contributed to lower costs in the direct discharge pathway (Table 5). The average costs of care were around double in the inpatient transfer pathway which had the highest proportions of severely disabled survivors.

**Table 5.** Average costs of inpatient and community-based stroke care costs per pathway.

Pathway	Route	No. of patients	Average inpatient cost/patient (£)^a^	Average community therapy cost/patient (£)^a^	Total average cost/patient (£)
Direct discharge pathway	R1	21,646	5461.4	0.0	5461.4
	R2	19,779	5300.8	0.0	5300.8
Community rehabilitation pathway	R3	17,308	4617.0	465.8	5082.8
	R4	17,250	4646.1	379.1	5025.2
Inpatient transfer pathway	R5	8410	10,601.7	914.8	11,516.5
	R6	1810	11,774.6	955.9	12,730.5
	R7	6479	11,239.1	0.0	11,239.1
	R8	1067	12,515.4	0.0	12,515.4
Other pathways	R9	1155	5769.8	972.6	6742.4

The shaded area denotes the routes which are common to two pathways.

## Discussion

The results of this study show that the routes that patients take through stroke services from initial admission to discharge from specialist stroke service routes are extremely varied, with over 800 different routes identified. However, we were able to consolidate these into nine summary routes and three pathways with common characteristics. These highlighted key ways of configuring overarching stroke services. We have characterized the patients who followed each route, their therapy, outcomes and the costs of inpatient care. To our knowledge, this is the first attempt to map out stroke services in this way. We believe it provides a useful framework to conceptualize the way stroke services are organized and the information it contains can be used to plan, benchmark and develop stroke services. The length of stay could be particularly useful as a benchmark to estimate expected discharge dates for patients treated by services with as similar configuration.

Many of the observed differences between the pathways could be explained by differences in the profile of stroke severity: those at the extremes of stroke severity were more likely to follow the direct discharge pathway and less likely to need therapy. Unsurprisingly, the length of stay was short on the direct discharge pathway as it involved a relatively high proportion of patients with very/mild stroke who were discharged quickly and also patients who died. Despite this, the cost of inpatient care was not lower on the direct discharge pathway than on Routes 4 and 5 (community rehabilitation after discharge from admitting team).

Consideration needs to be given to the high proportion of patients with mild stroke who were discharged without any community rehabilitation. There is growing recognition that people with ‘mild’ strokes often suffer enduring impairments but the limitations they impose on activity and participation only become apparent once patients are discharged and attempt to function within their own environment.^18–20^ This highlights the need for all stroke survivors to be actively monitored and supported after discharge with easy, rapid access to rehabilitation services when needed.

Patients who followed the community rehabilitation pathway tended to have, on average, milder strokes than the other routes. However, the demand for therapy was high and patients (who needed it) received relatively large amounts of therapy, even as an inpatient (Table 3). The differences in inpatient length of stay between routes with or without community rehabilitation were surprising. One of the big advantages of community rehabilitation, particularly early supported discharge, is said to be a reduced length of inpatient stay and thus costs (which are driven by length of stay).^4,5,21^ Thus, one might expect the length of inpatient stay and costs of the community rehabilitation pathway to be less than the other pathways. This was not the case; length of stay was similar (if discharged from the admitting team – Routes 3 and 4) or longer (if transferred to another inpatient team – Routes 5 and 6) for those who received community rehabilitation. This may be because patients who did not receive community rehabilitation tended to more frequently be disabled before their stroke and therefore may already have facilities in place to provide care. Second, as premorbid disability and severe stroke are predictors of poor recovery after stroke,^22^ these patients may not have been referred for community rehabilitation as they were thought to have little potential for further recovery.^23^ Alternatively, length of inpatient stay may have been longer for patients receiving community rehabilitation because of delays initiating community rehabilitation and/or care packages.

Routes involving initial admission to a combined stroke team had a longer median length of inpatient stay than those admitted to an acute stroke team in all three pathways. There were, however, no obvious differences in the patients’ demographics, stroke characteristics, therapy needs/provision or mortality to explain this. It suggests that whether a patient is treated by an acute or combined team is not driven by the patients’ needs or clinical decision-making but by managerial choices about how stroke teams are configured. Further research to investigate the causes of the difference in length of stay and to compare other outcomes such as quality of care, satisfaction and cost-effectiveness is warranted to see whether the different configurations are equivalent. Providing all inpatient stroke care in a combined acute and rehabilitation unit may be preferable as it could prevent potential delays and disruption caused by transfer between units, but a specific rehabilitation unit which is separate from the acute stroke unit may enable patients to receive more therapy without distraction from the demands for rapid assessment and discharge which are given priority in acute stroke care. In a recent paper, we have shown that conditional on needing therapy, the amount of therapy provided was associated with inpatient length of stay and thus resource use (more therapy was associated with lower resource use/cost).^21^ Thus, further research is merited to assess whether a configuration with separate acute and rehabilitation stroke units may prove more cost-effective. More broadly, further research is needed to investigate the costs and cost-effectiveness of different configurations of overarching stroke services and to understand the impact of rehabilitation on the use of NHS resources.

### Limitations

This work is based on routinely collected observational data which come with limitations that should be noted. Although SSNAP has stringent quality control processes, it is dependent on the accuracy of the original data entered and may therefore be open to observer and reporter bias. However, in a sample of this size, the impact of any such inaccuracy would be expected to be negligible and random. Furthermore, although we are confident of the validity of the classifications employed here, SSNAP focuses on stroke admission and hyper-acute care when recording information and classifying the types of stroke team. Postacute care receives less attention. We therefore needed to build on standard SSNAP terminology to characterize the patients’ inpatient locations and care pathways.^24^ For example, SSNAP provides useful in-house classifications for the type of admitting stroke team, but the team might cover any combination of hyper-acute care, acute care and/or rehabilitation. We have attempted to define these in more detail in a manner that is resonant with clinical care. Some misclassification may be present however. Although our classification has the advantage that it is driven by the data and clinical expertise, and has face validity, we understand any future work would be greatly improved if the exact type of stroke team in which the patient is located could be designated to reflect the care received by the patient throughout the care pathway (e.g. an hyper-acute; acute; combined or rehabilitation unit).

Our analyses were limited by the variables collected by SSNAP. Further research is needed using more detailed measures of patient-related outcomes and quality of care which were not possible with the information available. Furthermore, we limited our analysis to patients who were likely candidates for therapy by excluding patients who were discharged or died very soon after admission (within three days). Thus, the pathways do not represent all stroke patients. However, we would expect nearly all the patients who met our exclusion criteria would have followed the direct discharge pathway, so it is likely that although the proportion of patients and their characteristics in this pathway may change, the number or nature of the pathways would not.

Our data were collected from July 2013 to July 2015, which was a period of change in UK stroke services with many being re-organized to deliver hyper-acute care and specialist community services. Consequently, some stroke teams may have changed classification during the study period. To prevent possible patient identification, the exact date of admission was not available and so the classification designated by SSNAP at the mid-point of the study (June 2014) was applied. To reduce misclassification, an experienced member of the SSNAP team was consulted and the definitions we produced were vetted; however, misclassification may still be present.

Finally, as observational data were used, the results cannot be interpreted as a suggestion of causation for any differences observed, nor that one pathway is more cost-effective or represents an optimal use of resources. Prospective research, preferably involving random allocation and data collection for cost-effectiveness analysis, is required to address how best to configure services.

**Clinical messages**

Nine distinct routes describing three overarching stroke pathways were identified.

- Direct discharge (44%, with a high proportion of patients with very/severe and mild strokes);
- Inpatient transfer pathway (19% involving the most severe strokes who needed further inpatient rehabilitation);
- Community rehabilitation pathway (47% with the mildest stroke but high therapy demand);
- An ‘other’ pathway (1%).

## Supplemental Material

Online_Appendix – Supplemental material for How do patients pass through stroke services? Identifying stroke care pathways using national audit data Click here for additional data file. Supplemental material, Online_Appendix for How do patients pass through stroke services? Identifying stroke care pathways using national audit data by Matthew Gittins, David G Lugo-Palacios, Lizz Paley, Benjamin Bray, Audrey Bowen, Andy Vail, Brenda Gannon and Sarah Tyson in Clinical Rehabilitation

Supplementary_Data_Costs_by_pathway_and_severity – Supplemental material for How do patients pass through stroke services? Identifying stroke care pathways using national audit data Click here for additional data file. Supplemental material, Supplementary_Data_Costs_by_pathway_and_severity for How do patients pass through stroke services? Identifying stroke care pathways using national audit data by Matthew Gittins, David G Lugo-Palacios, Lizz Paley, Benjamin Bray, Audrey Bowen, Andy Vail, Brenda Gannon and Sarah Tyson in Clinical Rehabilitation
