# Supplementary material for: How do patients pass through stroke services? Identifying stroke care pathways using national audit data
Source: Clin Rehabil. 2020 Mar 6;34(5):698–709. doi: 10.1177/0269215520907654 (PMC7443957; doi:10.1177/0269215520907654)
Supplement: Online_Appendix – Supplemental material for How do patients pass through stroke services? Identifying stroke care pathways using national audit data [file Online_Appendix.pdf]

Appendix: All identified routes.

Key to locations

RATa: Routinely Admitting Team with median length of stay ≤7 days
RATc: Routinely Admitting Team with median length of stay >7 days
NRAT: Non-Routinely Admitting Team
NAIT: Non Acute Inpatient Team
Home: Patient's own home
ESD/CRT: Early Supported Discharge / Community Rehab Team
Non-SSNAPESD/CRT: Non-SSNAP ESD/CRT
Non-SSNAPIn: Non-SSNAP Inpatient team (possibly a general inpatient ward)
"+E": discharged with an ESD team
"+C": discharged with a CRT team
"+EC": discharged with both a ESD and a CRT team

Frequency:- Route
9520:- RATa; Home
9088:- RATc; Home
5853:- RATa; Died
5393:- RATc; Died
4838:- RATc; Home+C
3741:- RATa; Carehome
3603:- RATc; Carehome
3541:- RATa; Home+C
2813:- RATa; ESD/CRT+E; Home
2630:- RATc; ESD/CRT+E; Home
2589:- RATa; Home+E
2416:- RATc; Home+E
1738:- RATc; Home+EC
1672:- RATa; Elsewhere
1263:- RATa; NRAT; Home
1194:- RATc; Elsewhere
1147:- RATa; Home+EC
1049:- RATa; NRAT; Home+C
951:- RATa; NRAT; Died
944:- RATc; Carehome+C
904:- RATa; Elsewhere+C
867:- RATa; ESD/CRT+C; Home
849:- RATa; ESD/CRT+EC; Home
839:- RATa; Non-SSNAPIn
750:- RATa; NRAT; Carehome
615:- RATa; Carehome+C
601:- RATa; ESD/CRT+E
558:- RATc; ESD/CRT+C; Home
549:- RATc; ESD/CRT+EC; Home
548:- RATa; NAIT; Home+C
542:- RATa; NAIT; ESD/CRT+E; Home
537:- RATc; Elsewhere+C
468:- RATa; NAIT; Carehome
466:- RATc; Non-SSNAPIn

433:- RATa; NAIT; Home
420:- RATc; ESD/CRT+E
418:- RATc; Carehome+E
417:- RATa; NRAT; ESD/CRT+E; Home
412:- RATa; ESD/CRT+C
401:- RATa; NRAT; Elsewhere
337:- RATa; Non-SSNAPESD/CRT+E
300:- RATa; NRAT; ESD/CRT+C; Home
300:- RATa; Non-SSNAPIn+C
290:- RATa; RAT; Home
277:- RATa; NAIT; ESD/CRT+C; Home
269:- RATa; NRAT; Home+E
264:- RATc; NAIT; Home
259:- RATc; NAIT; Home+C
245:- RATa; ESD/CRT+EC
235:- RATa; RAT; Died
230:- RATa; ESD/CRT+E; Non-SSNAPESD/CRT
228:- RATc; Non-SSNAPIn+C
222:- RATc; ESD/CRT+C
220:- RATc; NAIT; ESD/CRT+E; Home
215:- RATa; Carehome+EC
214:- RATa; RAT; ESD/CRT+E; Home
204:- RATc; Carehome+EC
201:- RATa; Non-SSNAPESD/CRT+C
201:- RATc; NAIT; ESD/CRT+C; Home
199:- RATa; Elsewhere+E
194:- RATa; RAT; Carehome
192:- RATa; Carehome+E
186:- RATa; NRAT; Carehome+C
186:- RATa; NRAT; Home+EC
176:- RATa; NAIT; Elsewhere
175:- RATa; NAIT; Died
166:- RATa; RAT; Home+C
165:- RATc; NAIT; Carehome
157:- RATc; ESD/CRT+E; Carehome
153:- RATc; Non-SSNAPESD/CRT+E
149:- RATa; NAIT; Carehome+C
149:- RATa; NRAT; ESD/CRT+E; ESD/CRT+C; Home
148:- RATa; NAIT; ESD/CRT+E
147:- NRATc; Died
144:- RATa; NRAT; NRAT; Home
138:- RATa; NAIT; Home+E
137:- NRATc; Home
134:- RATc; ESD/CRT+E; ESD/CRT+C; Home
129:- RATa; NRAT; ESD/CRT+EC; Home
129:- RATc; ESD/CRT+EC
127:- RATc; RAT; Home+C
126:- RATa; Elsewhere+EC
125:- RATc; Elsewhere+E
124:- RATa; NAIT; ESD/CRT+EC; Home
124:- RATa; NRAT; NRAT; Carehome

122:- RATa; NRAT; Elsewhere+C
117:- RATa; NRAT; NRAT; Died
115:- RATa; NAIT; ESD/CRT+C
108:- RATa; ESD/CRT+E; Elsewhere
108:- RATa; NRAT; Non-SSNAPIn
107:- RATa; NRAT; NAIT; ESD/CRT+C; Home
106:- RATc; RAT; Home
102:- RATc; Non-SSNAPESD/CRT+C
101:- NRATc; Home+C
96:- RATa; ESD/CRT+EC; Non-SSNAPESD/CRT
93:- RATa; NRAT; ESD/CRT+E; ESD/CRT+EC; Home
93:- RATc; Elsewhere+EC
91:- RATc; NAIT; ESD/CRT+EC; Home
88:- RATa; NAIT; Non-SSNAPIn
85:- RATa; NRAT; NRAT; Home+C
83:- RATa; Non-SSNAPESD/CRT+EC
82:- RATa; NRAT; ESD/CRT+E
81:- NRATc; Carehome
79:- RATa; NRAT; ESD/CRT+C
79:- RATc; ESD/CRT+E; Elsewhere
77:- RATa; NRAT; ESD/CRT+EC
75:- RATa; ESD/CRT+C; Carehome
70:- RATa; RAT; Home+E
70:- RATc; NAIT; Died
69:- RATa; ESD/CRT+E; Carehome
69:- RATa; NAIT; ESD/CRT+E; Carehome
69:- RATc; RAT; Carehome
68:- RATc; ESD/CRT+E; Died
66:- RATa; ESD/CRT+E; ESD/CRT+C; Home
66:- RATc; ESD/CRT+C; Carehome
65:- RATa; NRAT; NAIT; Home+C
65:- RATa; RAT; Elsewhere
64:- RATa; NRAT; NRAT; Elsewhere
61:- RATc; NRAT; Home+C
61:- RATc; Non-SSNAPESD/CRT+EC
59:- RATa; NRAT; NAIT; Carehome
58:- NRATc; ESD/CRT+C; Home
57:- RATa; NRAT; NRAT; ESD/CRT+C; Home
57:- RATc; ESD/CRT+E; Non-SSNAPESD/CRT
56:- NRATc; Elsewhere
56:- RATc; NAIT; ESD/CRT+C
56:- RATc; NAIT; Home+EC
54:- RATc; RAT; Died
53:- NRATc; ESD/CRT+E; Home
53:- RATa; NRAT; Non-SSNAPESD/CRT+C
52:- RATa; NAIT; Home+EC
51:- RATa; RAT; ESD/CRT+E; ESD/CRT+C; Home
51:- RATa; Non-SSNAPIn+E
48:- RATa; NAIT; ESD/CRT+C; Carehome
46:- RATa; NRAT; NAIT; Home
46:- RATa; NRAT; Non-SSNAPESD/CRT+E

46:- RATc; NRAT; Carehome
45:- RATa; NAIT; Non-SSNAPESD/CRT+C
45:- RATa; NAIT; Elsewhere+C
45:- RATa; NRAT; NRAT; Home+EC
43:- RATa; NRAT; NRAT; ESD/CRT+E; Home
43:- RATc; NAIT; Elsewhere
42:- RATa; NAIT; Elsewhere+E
41:- RATc; NAIT; Carehome+C
41:- RATc; NAIT; Non-SSNAPIn
40:- RATa; NAIT; ESD/CRT+EC
40:- RATa; NRAT; ESD/CRT+E; ESD/CRT+C
39:- RATa; ESD/CRT+E; ESD/CRT+C
39:- RATa; RAT; ESD/CRT+C; Home
39:- RATc; NRAT; Died
38:- RATa; NRAT; Elsewhere+EC
38:- RATa; RAT; Non-SSNAPIn
37:- RATc; NAIT; ESD/CRT+E; Carehome
37:- RATc; NRAT; Home
37:- RATc; NRAT; Non-SSNAPIn
37:- RATc; RAT; ESD/CRT+E; Home
37:- RATc; Non-SSNAPIn+EC
36:- RATa; ESD/CRT+C; Died
36:- RATa; NRAT; Carehome+EC
36:- RATa; NRAT; Non-SSNAPESD/CRT+EC
36:- RATa; Non-SSNAPIn+EC
35:- RATa; ESD/CRT+E; ESD/CRT+EC; Home
35:- RATc; ESD/CRT+C; Died
35:- RATc; ESD/CRT+EC; Carehome
35:- RATc; RAT; Home+EC
35:- RATc; Non-SSNAPESD/CRT
34:- RATa; ESD/CRT+E; Died
34:- RATa; ESD/CRT+EC; Carehome
34:- RATa; RAT; Home+EC
33:- RATa; NRAT; Non-SSNAPIn+EC
33:- RATc; NRAT; ESD/CRT+C; Home
32:- NAIT; Home
32:- RATc; ESD/CRT+EC; Elsewhere
31:- RATa; RAT; ESD/CRT+E
31:- RATc; NRAT; ESD/CRT+E; Home
31:- RATc; NRAT; ESD/CRT+EC; Home
31:- RATc; Non-SSNAPIn+E
30:- RATa; NAIT; ESD/CRT+E; Elsewhere
30:- RATa; NRAT; ESD/CRT+EC; ESD/CRT+EC; Home
29:- RATa; NRAT; NAIT; ESD/CRT+C
28:- NRATc; Home+E
28:- RATa; NRAT; ESD/CRT+C; Carehome
27:- NRATc; Carehome+C
27:- RATa; NAIT; Carehome+EC
27:- RATa; NRAT; NAIT; Non-SSNAPIn
27:- RATc; NRAT; Carehome+C
26:- RATa; NRAT; ESD/CRT+E; Elsewhere

26:- RATa; NRAT; NRAT; ESD/CRT+EC; Home
26:- RATc; NRAT; ESD/CRT+C
25:- RATa; ESD/CRT+C; Elsewhere
25:- RATa; NRAT; ESD/CRT+C; Died
25:- RATa; NRAT; Non-SSNAPIn+C
25:- RATa; RAT; Carehome+C
25:- RATa; RAT; NAIT; ESD/CRT+E; Home
25:- RATa; RAT; Elsewhere+C
25:- RATc; NAIT; ESD/CRT+EC
24:- RATa; NAIT; ESD/CRT+E; ESD/CRT+C
23:- RATa; NAIT; Carehome+E
23:- RATa; NRAT; NRAT; ESD/CRT+E
23:- RATa; RAT; ESD/CRT+EC; Home
23:- RATc; NRAT; Non-SSNAPESD/CRT+C
23:- RATc; RAT; Elsewhere
22:- NRATc; Home+EC
22:- RATa; NRAT; ESD/CRT+E; ESD/CRT+EC
22:- RATa; NRAT; NRAT; Carehome+C
22:- RATc; RAT; Home+E
21:- NRATc; ESD/CRT+E; ESD/CRT+C; Home
21:- RATa; ESD/CRT+EC; ESD/CRT+EC
21:- RATa; RAT; ESD/CRT+E; Carehome
21:- RATa; Non-SSNAPESD/CRT
21:- RATc; ESD/CRT+C; Elsewhere
21:- RATc; NAIT; ESD/CRT+C; Carehome
21:- RATc; RAT; Carehome+C
20:- NAIT; Died
20:- RATa; NAIT; ESD/CRT+E; Non-SSNAPESD/CRT
20:- RATc; ESD/CRT+E; ESD/CRT+C
19:- RATa; ESD/CRT+EC; Died
19:- RATa; NRAT; NRAT; Home+E
19:- RATc; ESD/CRT+E; ESD/CRT+EC
18:- NRATc; ESD/CRT+EC; Home
18:- RATa; NRAT; ESD/CRT+C; Elsewhere
17:- NAIT; ESD/CRT+C; Home
17:- RATa; ESD/CRT+EC; Elsewhere
17:- RATa; NAIT; Elsewhere+EC
17:- RATa; NRAT; NAIT; ESD/CRT+C; Carehome
17:- RATa; NRAT; NAIT; Non-SSNAPESD/CRT+C
17:- RATa; NRAT; Elsewhere+E
17:- RATa; RAT; NAIT; Home+C
17:- RATc; ESD/CRT+EC; Died
17:- RATc; ESD/CRT+EC; Non-SSNAPESD/CRT
17:- RATc; NAIT; Home+E
16:- RATa; NAIT; ESD/CRT+EC; Non-SSNAPESD/CRT
16:- RATa; NRAT; NRAT; Carehome+EC
16:- RATa; RAT; ESD/CRT+E; Non-SSNAPESD/CRT
15:- RATa; ESD/CRT+C; Non-SSNAPESD/CRT
15:- RATa; NAIT; ESD/CRT+C; Died
15:- RATa; NAIT; Non-SSNAPESD/CRT+E
15:- RATa; NRAT; ESD/CRT+E; Carehome

15:- RATa; NRAT; NRAT; ESD/CRT+C
15:- RATa; NRAT; NRAT; ESD/CRT+E; ESD/CRT+C; Home
15:- RATa; NRAT; RAT; Died
15:- RATc; NAIT; Carehome+EC
15:- RATc; NAIT; Elsewhere+E
14:- RATa; NRAT; NAIT; Elsewhere
14:- RATa; NRAT; NRAT; Non-SSNAPIn
14:- RATa; RAT; ESD/CRT+C
14:- RATa; RAT; ESD/CRT+C; Carehome
14:- RATc; ESD/CRT+E; ESD/CRT+EC; Home
13:- RATa; ESD/CRT+EC; ESD/CRT+EC; Home
13:- RATa; NAIT; ESD/CRT+C; Elsewhere
13:- RATa; NRAT; NAIT; Died
13:- RATa; RAT; Carehome+E
13:- RATa; RAT; ESD/CRT+E; Died
13:- RATa; RAT; ESD/CRT+E; Elsewhere
13:- RATc; RAT; Elsewhere+C
12:- RATa; NRAT; ESD/CRT+EC; ESD/CRT+C; Home
12:- RATa; NRAT; NAIT; Carehome+C
12:- RATa; NRAT; NAIT; ESD/CRT+E; ESD/CRT+EC; Home
12:- RATa; NRAT; NAIT; Home+EC
12:- RATa; NRAT; NRAT; ESD/CRT+EC
12:- RATa; NRAT; NRAT; Elsewhere+C
12:- RATa; NRAT; RAT; Home
12:- RATa; RAT; NAIT; Home
12:- RATc; ESD/CRT+EC; ESD/CRT+C; Home
11:- RATa; ESD/CRT+E; ESD/CRT+EC
11:- RATa; NAIT; ESD/CRT+E; ESD/CRT+C; Home
11:- RATa; NAIT; Non-SSNAPIn+C
11:- RATa; NRAT; ESD/CRT+E; Died
11:- RATa; NRAT; NRAT; NAIT; ESD/CRT+C; Home
11:- RATa; NRAT; NRAT; Elsewhere+EC
11:- RATa; RAT; Carehome+EC
11:- RATa; RAT; ESD/CRT+E; ESD/CRT+C
11:- RATa; RAT; NAIT; ESD/CRT+C; Home
11:- RATc; NAIT; Non-SSNAPESD/CRT+C
11:- RATc; NAIT; Elsewhere+C
11:- RATc; NRAT; Elsewhere
10:- NAIT; Home
10:- NRATc; Non-SSNAPIn
10:- NRATc; Elsewhere+C
10:- RATa; ESD/CRT+EC; ESD/CRT+C; Home
10:- RATa; NAIT; RAT; Died
10:- RATa; NRAT; Carehome+E
10:- RATa; NRAT; NAIT; ESD/CRT+C; Died
10:- RATa; NRAT; NRAT; ESD/CRT+E; ESD/CRT+C
10:- RATc; NAIT; ESD/CRT+C; Elsewhere
10:- RATc; RAT; ESD/CRT+E
9:- NAIT; Home+C
9:- NRATc; NAIT; Home+C
9:- NRATc; RAT; Home

9:- NRATc; RAT; NRAT; Died
9:- NRATc; Elsewhere+E
9:- RATa; NRAT; ESD/CRT+C; ESD/CRT+C; Home
9:- RATa; NRAT; NAIT; Elsewhere+C
9:- RATa; NRAT; NRAT; NAIT; Carehome
9:- RATa; NRAT; NRAT; Non-SSNAPESD/CRT+C
9:- RATa; RAT; RAT; Home
9:- RATa; RAT; Non-SSNAPESD/CRT+C
9:- RATc; ESD/CRT+C; ESD/CRT+C; Home
9:- RATc; NAIT; ESD/CRT+EC; Elsewhere
9:- RATc; RAT; RAT; Home
8:- NAIT; ESD/CRT+E; Home
8:- NRATc; NAIT; ESD/CRT+C; Home
8:- NRATc; RAT; NRAT; Home
8:- RATa; NRAT; ESD/CRT+E; ESD/CRT+C; Elsewhere
8:- RATa; NRAT; ESD/CRT+E; Non-SSNAPESD/CRT
8:- RATa; NRAT; ESD/CRT+EC; Died
8:- RATa; NRAT; ESD/CRT+EC; ESD/CRT+C
8:- RATa; NRAT; ESD/CRT+EC; Non-SSNAPESD/CRT
8:- RATa; NRAT; NRAT; ESD/CRT+E; ESD/CRT+EC; Home
8:- RATa; NRAT; RAT; Elsewhere
8:- RATa; RAT; ESD/CRT+C; Died
8:- RATa; RAT; NAIT; Carehome
8:- RATc; RAT; ESD/CRT+C; Home
7:- NAIT; Carehome
7:- NRATc; ESD/CRT+C
7:- NRATc; ESD/CRT+E
7:- NRATc; ESD/CRT+E; ESD/CRT+EC; Home
7:- NRATc; RAT; NRAT; Home+E
7:- RATa; NAIT; RAT; NAIT; ESD/CRT+C
7:- RATa; NRAT; NAIT; ESD/CRT+E; Home
7:- RATa; NRAT; NRAT; NAIT; Home+C
7:- RATa; NRAT; RAT; ESD/CRT+E; Home
7:- RATa; RAT; RAT; Non-SSNAPIn
7:- RATa; RAT; RAT; Elsewhere
7:- RATc; NAIT; ESD/CRT+E
7:- RATc; NAIT; RAT; Died
7:- RATc; RAT; ESD/CRT+EC; Home
7:- RATc; RAT; NAIT; ESD/CRT+E; Home
7:- RATc; RAT; NAIT; Home+C
7:- RATc; RAT; Non-SSNAPIn
6:- NAIT; Died
6:- NRATc; NRAT; Died
6:- NRATc; RAT; NRAT; Home+C
6:- NRATc; Non-SSNAPESD/CRT+E
6:- RATa; NAIT; ESD/CRT+E; Died
6:- RATa; NAIT; ESD/CRT+EC; Carehome
6:- RATa; NAIT; ESD/CRT+EC; Elsewhere
6:- RATa; NAIT; RAT; NAIT; Carehome
6:- RATa; NAIT; Non-SSNAPESD/CRT+EC
6:- RATa; NRAT; ESD/CRT+EC; ESD/CRT+EC

6:- RATa; NRAT; NAIT; ESD/CRT+C; Elsewhere
6:- RATa; NRAT; NAIT; Non-SSNAPESD/CRT+E
6:- RATa; NRAT; NRAT; ESD/CRT+EC; ESD/CRT+EC; Home
6:- RATa; NRAT; NRAT; NAIT; Home
6:- RATa; NRAT; NRAT; Non-SSNAPESD/CRT+EC
6:- RATa; NRAT; NRAT; Non-SSNAPIn+C
6:- RATa; NRAT; RAT; Home+E
6:- RATa; NRAT; Non-SSNAPIn+E
6:- RATa; RAT; ESD/CRT+E; ESD/CRT+EC; Home
6:- RATa; RAT; NAIT; ESD/CRT+EC; Home
6:- RATa; RAT; NAIT; Non-SSNAPESD/CRT+C
6:- RATa; RAT; RAT; Carehome
6:- RATa; RAT; RAT; Died
6:- RATa; RAT; Non-SSNAPESD/CRT+E
6:- RATa; RAT; Non-SSNAPIn+C
6:- RATc; ESD/CRT+E; ESD/CRT+C; Elsewhere
6:- RATc; NAIT; ESD/CRT+C; Died
6:- RATc; NAIT; ESD/CRT+E; Non-SSNAPESD/CRT
6:- RATc; RAT; ESD/CRT+E; Non-SSNAPESD/CRT
6:- RATc; RAT; NAIT; Home
6:- RATc; RAT; RAT; Home+C
5:- NAIT; ESD/CRT+E
5:- NRATc; ESD/CRT+E; ESD/CRT+C
5:- NRATc; RAT; NRAT; Home+EC
5:- RATa; ESD/CRT+C; ESD/CRT+C; Home
5:- RATa; NAIT; NAIT; Home+C
5:- RATa; NAIT; RAT; Home
5:- RATa; NAIT; RAT; NAIT; Elsewhere
5:- RATa; NRAT; NAIT; ESD/CRT+EC; Home
5:- RATa; NRAT; NRAT; NAIT; ESD/CRT+C
5:- RATa; NRAT; NRAT; Non-SSNAPIn+EC
5:- RATa; NRAT; RAT; Carehome
5:- RATa; NRAT; RAT; Home+C
5:- RATa; RAT; NAIT; Carehome+C
5:- RATa; RAT; NAIT; ESD/CRT+C
5:- RATa; RAT; NAIT; ESD/CRT+E; Carehome
5:- RATa; RAT; RAT; Home+C
5:- RATa; RAT; RAT; Home+E
5:- RATc; RAT; NAIT; Carehome
5:- RATc; RAT; RAT; Carehome
5:- RATc; RAT; RAT; ESD/CRT+E; Home
5:- RATc; RAT; Elsewhere+EC
4:- NAIT; ESD/CRT+C
4:- NRATc; ESD/CRT+C; Died
4:- NRATc; ESD/CRT+EC; ESD/CRT+C; Home
4:- NRATc; NAIT; Elsewhere
4:- NRATc; RAT; NRAT; Carehome
4:- NRATc; Non-SSNAPESD/CRT+C
4:- RATa; NAIT; Non-SSNAPESD/CRT
4:- RATa; NRAT; ESD/CRT+EC; Elsewhere
4:- RATa; NRAT; NAIT; ESD/CRT+E; ESD/CRT+EC

4:- RATa; NRAT; NRAT; ESD/CRT+C; ESD/CRT+C; Home
4:- RATa; NRAT; NRAT; Non-SSNAPESD/CRT+E
4:- RATa; NRAT; Non-SSNAPESD/CRT
4:- RATa; RAT; NAIT; Died
4:- RATa; RAT; NAIT; Non-SSNAPIn
4:- RATa; RAT; RAT; ESD/CRT+C; Home
4:- RATa; RAT; RAT; ESD/CRT+E; Home
4:- RATa; RAT; Elsewhere+EC
4:- RATc; NAIT; ESD/CRT+EC; Carehome
4:- RATc; NAIT; Non-SSNAPIn+C
4:- RATc; NRAT; Non-SSNAPIn+C
4:- RATc; RAT; Carehome+E
4:- RATc; RAT; ESD/CRT+E; ESD/CRT+C; Home
4:- RATc; RAT; ESD/CRT+EC
4:- RATc; RAT; NAIT; Home+EC
4:- RATc; RAT; Non-SSNAPIn+EC
3:- NAIT; Elsewhere
3:- NAIT; Home+C
3:- NAIT; Home+E
3:- NRATc; Carehome+E
3:- NRATc; ESD/CRT+C; Carehome
3:- NRATc; NRAT; Home
3:- NRATc; RAT; NRAT; ESD/CRT+E; Home
3:- RATa; ESD/CRT+E; ESD/CRT+E; Home
3:- RATa; NAIT; ESD/CRT+C; ESD/CRT+EC
3:- RATa; NAIT; ESD/CRT+C; Non-SSNAPESD/CRT
3:- RATa; NAIT; ESD/CRT+E; ESD/CRT+EC; Home
3:- RATa; NAIT; ESD/CRT+EC; Died
3:- RATa; NAIT; NAIT; ESD/CRT+C; Home
3:- RATa; NAIT; RAT; NAIT; Died
3:- RATa; NAIT; RAT; NAIT; Home
3:- RATa; NAIT; RAT; Elsewhere
3:- RATa; NAIT; Non-SSNAPIn+E
3:- RATa; NRAT; ESD/CRT+E; ESD/CRT+C; Carehome
3:- RATa; NRAT; ESD/CRT+E; ESD/CRT+C; Died
3:- RATa; NRAT; ESD/CRT+EC; Carehome
3:- RATa; NRAT; NAIT; ESD/CRT+EC; ESD/CRT+EC; Home
3:- RATa; NRAT; NAIT; NAIT; Home+EC
3:- RATa; NRAT; NAIT; Non-SSNAPIn+C
3:- RATa; NRAT; NRAT; Carehome+E
3:- RATa; NRAT; NRAT; ESD/CRT+C; Carehome
3:- RATa; NRAT; NRAT; ESD/CRT+C; Died
3:- RATa; NRAT; NRAT; ESD/CRT+E; Elsewhere
3:- RATa; NRAT; NRAT; ESD/CRT+EC; ESD/CRT+C
3:- RATa; NRAT; RAT; ESD/CRT+C; Home
3:- RATa; NRAT; RAT; ESD/CRT+EC; Home
3:- RATa; NRAT; RAT; NRAT; Died
3:- RATa; NRAT; RAT; NRAT; ESD/CRT+EC
3:- RATa; RAT; NAIT; ESD/CRT+E; Non-SSNAPESD/CRT
3:- RATa; RAT; NAIT; Home+EC
3:- RATa; RAT; NAIT; Elsewhere

3:- RATa; RAT; NRAT; Died
3:- RATa; RAT; Elsewhere+E
3:- RATc; ESD/CRT+EC; ESD/CRT+EC; Home
3:- RATc; NAIT; Carehome+E
3:- RATc; NAIT; ESD/CRT+E; Elsewhere
3:- RATc; NAIT; RAT; NAIT; Home+C
3:- RATc; NAIT; Elsewhere+EC
3:- RATc; NRAT; ESD/CRT+C; Died
3:- RATc; RAT; ESD/CRT+C
3:- RATc; RAT; ESD/CRT+E; Carehome
3:- RATc; RAT; NAIT; ESD/CRT+C; Home
3:- RATc; RAT; RAT; Died
3:- RATc; RAT; RAT; ESD/CRT+E
3:- RATc; RAT; RAT; Elsewhere
3:- RATc; RAT; Non-SSNAPIn+C
2:- NAIT; Carehome
2:- NAIT; Home+E
2:- NAIT; Carehome+C
2:- NAIT; ESD/CRT+C; Carehome
2:- NRATa; ESD/CRT+EC; Home
2:- NRATa; NRAT; Home
2:- NRATa; RAT; NRAT; Carehome
2:- NRATc; Carehome+EC
2:- NRATc; ESD/CRT+E; Died
2:- NRATc; ESD/CRT+EC; ESD/CRT+C
2:- NRATc; NAIT; ESD/CRT+C
2:- NRATc; NAIT; Home
2:- NRATc; NRAT; Carehome
2:- NRATc; NRAT; Home+C
2:- NRATc; NRAT; Elsewhere+EC
2:- NRATc; RAT; ESD/CRT+E; Home
2:- NRATc; RAT; NRAT; Carehome+C
2:- NRATc; RAT; NRAT; Elsewhere+C
2:- NRATc; RAT; NRAT; Elsewhere+E
2:- NRATc; RAT; NRAT; Elsewhere
2:- NRATc; Non-SSNAPIn+C
2:- NRATc; Elsewhere+EC
2:- RATa; ESD/CRT+C; ESD/CRT+C; Carehome
2:- RATa; ESD/CRT+C; ESD/CRT+EC
2:- RATa; ESD/CRT+C; ESD/CRT+EC; Home
2:- RATa; ESD/CRT+E; ESD/CRT+C; Elsewhere
2:- RATa; ESD/CRT+E; ESD/CRT+E
2:- RATa; ESD/CRT+EC; ESD/CRT+C
2:- RATa; ESD/CRT+EC; ESD/CRT+EC; Died
2:- RATa; ESD/CRT+EC; ESD/CRT+EC; ESD/CRT+EC
2:- RATa; NAIT; ESD/CRT+C; ESD/CRT+C
2:- RATa; NAIT; ESD/CRT+C; ESD/CRT+C; Home
2:- RATa; NAIT; ESD/CRT+EC; ESD/CRT+EC; Home
2:- RATa; NAIT; NAIT; Carehome+C
2:- RATa; NAIT; RAT; NAIT; ESD/CRT+E
2:- RATa; NAIT; RAT; NAIT; ESD/CRT+E; ESD/CRT+C

2:- RATa; NAIT; RAT; NAIT; RAT; ESD/CRT+E
2:- RATa; NAIT; RAT; NAIT; RAT; NAIT; Carehome
2:- RATa; NAIT; Non-SSNAPIn+EC
2:- RATa; NRAT; ESD/CRT+E; ESD/CRT+E; Home
2:- RATa; NRAT; ESD/CRT+E; ESD/CRT+EC; Carehome
2:- RATa; NRAT; NAIT; Carehome+EC
2:- RATa; NRAT; NAIT; ESD/CRT+E; ESD/CRT+C; Home
2:- RATa; NRAT; NAIT; ESD/CRT+E; Non-SSNAPESD/CRT
2:- RATa; NRAT; NAIT; Home+E
2:- RATa; NRAT; NAIT; NAIT; ESD/CRT+C
2:- RATa; NRAT; NAIT; NAIT; Non-SSNAPIn
2:- RATa; NRAT; NAIT; Non-SSNAPESD/CRT+EC
2:- RATa; NRAT; NRAT; ESD/CRT+C; Elsewhere
2:- RATa; NRAT; NRAT; ESD/CRT+E; Carehome
2:- RATa; NRAT; NRAT; ESD/CRT+E; Died
2:- RATa; NRAT; NRAT; ESD/CRT+E; Non-SSNAPESD/CRT
2:- RATa; NRAT; NRAT; ESD/CRT+EC; ESD/CRT+EC
2:- RATa; NRAT; NRAT; NAIT; ESD/CRT+C; Carehome
2:- RATa; NRAT; NRAT; NAIT; Non-SSNAPESD/CRT+C
2:- RATa; NRAT; NRAT; NAIT; Non-SSNAPIn
2:- RATa; NRAT; NRAT; NAIT; Elsewhere
2:- RATa; NRAT; NRAT; NRAT; Non-SSNAPIn
2:- RATa; NRAT; NRAT; NRAT; Elsewhere+EC
2:- RATa; NRAT; NRAT; Elsewhere+E
2:- RATa; NRAT; RAT; Carehome+C
2:- RATa; NRAT; RAT; NAIT; ESD/CRT+C; Home
2:- RATa; NRAT; RAT; NAIT; Home+C
2:- RATa; NRAT; RAT; NRAT; Carehome
2:- RATa; NRAT; RAT; NRAT; NRAT; Died
2:- RATa; NRAT; RAT; Elsewhere+C
2:- RATa; RAT; ESD/CRT+C; Elsewhere
2:- RATa; RAT; ESD/CRT+E; ESD/CRT+C; Elsewhere
2:- RATa; RAT; ESD/CRT+E; ESD/CRT+E
2:- RATa; RAT; ESD/CRT+E; ESD/CRT+EC
2:- RATa; RAT; ESD/CRT+EC; Non-SSNAPESD/CRT
2:- RATa; RAT; NAIT; ESD/CRT+E
2:- RATa; RAT; NAIT; ESD/CRT+EC; Carehome
2:- RATa; RAT; NAIT; ESD/CRT+EC; Elsewhere
2:- RATa; RAT; NAIT; Home+E
2:- RATa; RAT; NAIT; Elsewhere+C
2:- RATa; RAT; NRAT; Home+C
2:- RATa; RAT; NRAT; Elsewhere+C
2:- RATa; RAT; RAT; Carehome+C
2:- RATa; RAT; RAT; ESD/CRT+E; Non-SSNAPESD/CRT
2:- RATa; RAT; RAT; ESD/CRT+EC; ESD/CRT+EC
2:- RATa; RAT; RAT; Home+EC
2:- RATa; RAT; RAT; NAIT; ESD/CRT+E; Home
2:- RATa; RAT; RAT; NRAT; Home+EC
2:- RATa; RAT; RAT; Non-SSNAPESD/CRT+E
2:- RATa; RAT; RAT; Non-SSNAPIn+C
2:- RATc; ESD/CRT+E; ESD/CRT+C; Carehome

2:- RATc; ESD/CRT+EC; ESD/CRT+C
2:- RATc; ESD/CRT+EC; ESD/CRT+EC
2:- RATc; NAIT; ESD/CRT+C; ESD/CRT+C
2:- RATc; NAIT; NAIT; ESD/CRT+C; Home
2:- RATc; NAIT; RAT; Carehome
2:- RATc; NAIT; RAT; Elsewhere
2:- RATc; NRAT; ESD/CRT+C; Carehome
2:- RATc; NRAT; ESD/CRT+C; Elsewhere
2:- RATc; NRAT; ESD/CRT+E
2:- RATc; NRAT; ESD/CRT+E; ESD/CRT+C; Home
2:- RATc; NRAT; ESD/CRT+EC
2:- RATc; NRAT; Home+EC
2:- RATc; NRAT; Non-SSNAPESD/CRT+EC
2:- RATc; NRAT; Non-SSNAPESD/CRT
2:- RATc; RAT; Carehome+EC
2:- RATc; RAT; ESD/CRT+E; Died
2:- RATc; RAT; ESD/CRT+EC; Carehome
2:- RATc; RAT; ESD/CRT+EC; Elsewhere
2:- RATc; RAT; NAIT; ESD/CRT+C; Carehome
2:- RATc; RAT; NAIT; ESD/CRT+E
2:- RATc; RAT; NAIT; ESD/CRT+EC; Home
2:- RATc; RAT; NAIT; Non-SSNAPESD/CRT+C
2:- RATc; RAT; NAIT; Non-SSNAPIn
2:- RATc; RAT; RAT; ESD/CRT+EC; Home
2:- RATc; RAT; RAT; Home+E
2:- RATc; RAT; RAT; Non-SSNAPIn
2:- RATc; RAT; RAT; Elsewhere+C
2:- RATc; RAT; Non-SSNAPESD/CRT+EC
2:- RATc; RAT; Non-SSNAPIn+E
1:- NAIT; Carehome+EC
1:- NAIT; Home+EC
1:- NAIT; RAT; Home+E
1:- NAIT; RAT; Home
1:- NAIT; Elsewhere+E
1:- NAIT; Elsewhere+EC
1:- NAIT; Carehome+EC
1:- NAIT; ESD/CRT+C; Elsewhere
1:- NAIT; ESD/CRT+E; Carehome
1:- NAIT; ESD/CRT+E; Died
1:- NAIT; ESD/CRT+E; ESD/CRT+EC; Home
1:- NAIT; ESD/CRT+E; Elsewhere
1:- NAIT; ESD/CRT+EC; Home
1:- NAIT; NRAT; NAIT; Home+C
1:- NAIT; RAT; Carehome
1:- NAIT; RAT; ESD/CRT+E; Elsewhere
1:- NAIT; RAT; Home
1:- NAIT; RAT; RAT; ESD/CRT+E; Home
1:- NAIT; Elsewhere+C
1:- NRATa; Carehome+EC
1:- NRATa; Carehome
1:- NRATa; Died

1:- NRATa; ESD/CRT+EC
1:- NRATa; ESD/CRT+EC; Elsewhere
1:- NRATa; Home+E
1:- NRATa; Home
1:- NRATa; NRAT; Carehome+C
1:- NRATa; NRAT; ESD/CRT+E; ESD/CRT+EC
1:- NRATa; NRAT; ESD/CRT+E; Home
1:- NRATa; NRAT; ESD/CRT+EC; Elsewhere
1:- NRATa; NRAT; Home+C
1:- NRATa; NRAT; Home+E
1:- NRATa; NRAT; Non-SSNAPESD/CRT+EC
1:- NRATa; NRAT; Elsewhere
1:- NRATa; RAT; NRAT; ESD/CRT+E; Home
1:- NRATa; RAT; NRAT; ESD/CRT+EC; Elsewhere
1:- NRATa; RAT; NRAT; Home
1:- NRATa; RAT; NRAT; RAT; NRAT; Died
1:- NRATa; RAT; Non-SSNAPIn
1:- NRATa; Elsewhere
1:- NRATc; ESD/CRT+C; ESD/CRT+EC; Home
1:- NRATc; ESD/CRT+C; Non-SSNAPESD/CRT
1:- NRATc; ESD/CRT+C; Elsewhere
1:- NRATc; ESD/CRT+E; ESD/CRT+EC
1:- NRATc; ESD/CRT+E; Elsewhere
1:- NRATc; ESD/CRT+EC
1:- NRATc; ESD/CRT+EC; ESD/CRT+EC; Home
1:- NRATc; NAIT; Carehome
1:- NRATc; NAIT; ESD/CRT+E; ESD/CRT+C
1:- NRATc; NAIT; ESD/CRT+E; Home
1:- NRATc; NRAT; ESD/CRT+C; Carehome
1:- NRATc; NRAT; ESD/CRT+C; Home
1:- NRATc; NRAT; ESD/CRT+E; ESD/CRT+C
1:- NRATc; NRAT; ESD/CRT+EC; ESD/CRT+C; Died
1:- NRATc; NRAT; ESD/CRT+EC; Home
1:- NRATc; NRAT; Home+EC
1:- NRATc; NRAT; Elsewhere+E
1:- NRATc; NRAT; Elsewhere
1:- NRATc; RAT; Died
1:- NRATc; RAT; ESD/CRT+C; Carehome
1:- NRATc; RAT; ESD/CRT+E
1:- NRATc; RAT; Home+C
1:- NRATc; RAT; NAIT; ESD/CRT+C; Home
1:- NRATc; RAT; NRAT; Carehome+EC
1:- NRATc; RAT; NRAT; ESD/CRT+C; Died
1:- NRATc; RAT; NRAT; ESD/CRT+C; Home
1:- NRATc; RAT; NRAT; ESD/CRT+E; ESD/CRT+C; Home
1:- NRATc; RAT; NRAT; ESD/CRT+EC; Home
1:- NRATc; RAT; NRAT; NAIT; Home+C
1:- NRATc; RAT; NRAT; NRAT; Home+E
1:- NRATc; RAT; RAT; ESD/CRT+E; Home
1:- NRATc; RAT; Non-SSNAPESD/CRT+E
1:- NRATc; Non-SSNAPESD/CRT+EC

1:- NRATc; Non-SSNAPESD/CRT
1:- NRATc; Non-SSNAPIn+EC
1:- RATa; ESD/CRT+C; ESD/CRT+C; ESD/CRT+EC; Home
1:- RATa; ESD/CRT+C; ESD/CRT+C; Elsewhere
1:- RATa; ESD/CRT+E; ESD/CRT+C; ESD/CRT+C; Home
1:- RATa; ESD/CRT+E; ESD/CRT+C; ESD/CRT+E
1:- RATa; ESD/CRT+E; ESD/CRT+E; Elsewhere
1:- RATa; ESD/CRT+E; ESD/CRT+EC; Died
1:- RATa; ESD/CRT+EC; ESD/CRT+EC; Elsewhere
1:- RATa; Home+C; ESD/CRT; Home
1:- RATa; NAIT; ESD/CRT+C; ESD/CRT+E
1:- RATa; NAIT; ESD/CRT+C; ESD/CRT+EC; Home
1:- RATa; NAIT; ESD/CRT+E; ESD/CRT+C; Carehome
1:- RATa; NAIT; ESD/CRT+E; ESD/CRT+C; Died
1:- RATa; NAIT; ESD/CRT+E; ESD/CRT+C; Non-SSNAPESD/CRT
1:- RATa; NAIT; ESD/CRT+E; ESD/CRT+EC
1:- RATa; NAIT; ESD/CRT+EC; ESD/CRT+C
1:- RATa; NAIT; ESD/CRT+EC; ESD/CRT+C; Home
1:- RATa; NAIT; NAIT; Carehome+EC
1:- RATa; NAIT; NAIT; Carehome
1:- RATa; NAIT; NAIT; Died
1:- RATa; NAIT; NAIT; ESD/CRT+E; Home
1:- RATa; NAIT; NAIT; ESD/CRT+EC
1:- RATa; NAIT; NAIT; Home+EC
1:- RATa; NAIT; NAIT; Home
1:- RATa; NAIT; NAIT; RAT; Home
1:- RATa; NAIT; NRAT; Died
1:- RATa; NAIT; RAT; Carehome+E
1:- RATa; NAIT; RAT; Carehome
1:- RATa; NAIT; RAT; ESD/CRT+C; Carehome
1:- RATa; NAIT; RAT; ESD/CRT+C; Home
1:- RATa; NAIT; RAT; ESD/CRT+E; Home
1:- RATa; NAIT; RAT; Home+EC
1:- RATa; NAIT; RAT; NAIT; ESD/CRT+E; Home
1:- RATa; NAIT; RAT; NAIT; RAT; NAIT; ESD/CRT+C
1:- RATa; NAIT; RAT; NAIT; RAT; NAIT; RAT; NAIT; CarehomeN
1:- RATa; NAIT; RAT; NRAT; NAIT; ESD/CRT+C
1:- RATa; NAIT; RAT; NRAT; NAIT; Home
1:- RATa; NAIT; RAT; Non-SSNAPIn+C
1:- RATa; NRAT; ESD/CRT+C; ESD/CRT+C; Elsewhere
1:- RATa; NRAT; ESD/CRT+C; ESD/CRT+E; Home
1:- RATa; NRAT; ESD/CRT+C; Non-SSNAPESD/CRT
1:- RATa; NRAT; ESD/CRT+E; ESD/CRT+C; ESD/CRT+C
1:- RATa; NRAT; ESD/CRT+E; ESD/CRT+C; Non-SSNAPESD/CRT
1:- RATa; NRAT; ESD/CRT+EC; ESD/CRT+C; Non-SSNAPESD/CRT
1:- RATa; NRAT; ESD/CRT+EC; ESD/CRT+C; Elsewhere
1:- RATa; NRAT; ESD/CRT+EC; ESD/CRT+EC; Died
1:- RATa; NRAT; Home; ESD/CRT; Home
1:- RATa; NRAT; NAIT; ESD/CRT+C; ESD/CRT+C; Home
1:- RATa; NRAT; NAIT; ESD/CRT+C; ESD/CRT+E
1:- RATa; NRAT; NAIT; ESD/CRT+E

1:- RATa; NRAT; NAIT; ESD/CRT+E; ESD/CRT+C
1:- RATa; NRAT; NAIT; ESD/CRT+E; Elsewhere
1:- RATa; NRAT; NAIT; ESD/CRT+EC; ESD/CRT+EC
1:- RATa; NRAT; NAIT; ESD/CRT+EC; Elsewhere
1:- RATa; NRAT; NAIT; NAIT; Carehome+C
1:- RATa; NRAT; NAIT; NAIT; Carehome
1:- RATa; NRAT; NAIT; NAIT; ESD/CRT+C; Home
1:- RATa; NRAT; NAIT; NAIT; Home+C
1:- RATa; NRAT; NAIT; NRAT; Carehome+C
1:- RATa; NRAT; NAIT; NRAT; Carehome
1:- RATa; NRAT; NAIT; RAT; Carehome+C
1:- RATa; NRAT; NAIT; RAT; Carehome+E
1:- RATa; NRAT; NAIT; RAT; Died
1:- RATa; NRAT; NAIT; RAT; Home
1:- RATa; NRAT; NAIT; RAT; NAIT; Carehome+EC
1:- RATa; NRAT; NAIT; RAT; NAIT; Home
1:- RATa; NRAT; NAIT; RAT; NRAT; NAIT; Elsewhere
1:- RATa; NRAT; NRAT; ESD/CRT+C; Non-SSNAPESD/CRT
1:- RATa; NRAT; NRAT; ESD/CRT+EC; ESD/CRT+C; Home
1:- RATa; NRAT; NRAT; ESD/CRT+EC; ESD/CRT+EC; Carehome
1:- RATa; NRAT; NRAT; ESD/CRT+EC; Non-SSNAPESD/CRT
1:- RATa; NRAT; NRAT; NAIT; Carehome+C
1:- RATa; NRAT; NRAT; NAIT; ESD/CRT+E; ESD/CRT+EC; Home
1:- RATa; NRAT; NRAT; NAIT; ESD/CRT+E; Home
1:- RATa; NRAT; NRAT; NAIT; ESD/CRT+EC; Home
1:- RATa; NRAT; NRAT; NAIT; Home+EC
1:- RATa; NRAT; NRAT; NAIT; Non-SSNAPESD/CRT+EC
1:- RATa; NRAT; NRAT; NAIT; Non-SSNAPIn+C
1:- RATa; NRAT; NRAT; NRAT; Carehome+E
1:- RATa; NRAT; NRAT; NRAT; Died
1:- RATa; NRAT; NRAT; NRAT; ESD/CRT+C; ESD/CRT+C
1:- RATa; NRAT; NRAT; NRAT; ESD/CRT+E; ESD/CRT+EC; Home
1:- RATa; NRAT; NRAT; NRAT; Home+C
1:- RATa; NRAT; NRAT; NRAT; Home+EC
1:- RATa; NRAT; NRAT; NRAT; Non-SSNAPIn+EC
1:- RATa; NRAT; NRAT; RAT; Died
1:- RATa; NRAT; NRAT; RAT; NRAT; ESD/CRT+E; Home
1:- RATa; NRAT; NRAT; RAT; NRAT; Non-SSNAPIn
1:- RATa; NRAT; RAT; Carehome+EC
1:- RATa; NRAT; RAT; ESD/CRT+C
1:- RATa; NRAT; RAT; ESD/CRT+C; Died
1:- RATa; NRAT; RAT; ESD/CRT+E; Carehome
1:- RATa; NRAT; RAT; ESD/CRT+E; ESD/CRT+C; Non-SSNAPESD/CRT
1:- RATa; NRAT; RAT; ESD/CRT+EC; Non-SSNAPESD/CRT
1:- RATa; NRAT; RAT; Home+EC
1:- RATa; NRAT; RAT; NAIT; Carehome+C
1:- RATa; NRAT; RAT; NAIT; Carehome+EC
1:- RATa; NRAT; RAT; NAIT; ESD/CRT+E; Home
1:- RATa; NRAT; RAT; NAIT; ESD/CRT+EC; Home
1:- RATa; NRAT; RAT; NAIT; Home+EC
1:- RATa; NRAT; RAT; NAIT; Home

1:- RATa; NRAT; RAT; NAIT; NAIT; Carehome
1:- RATa; NRAT; RAT; NAIT; Non-SSNAPIn+EC
1:- RATa; NRAT; RAT; NAIT; Elsewhere+C
1:- RATa; NRAT; RAT; NRAT; ESD/CRT+C
1:- RATa; NRAT; RAT; NRAT; ESD/CRT+C; Home
1:- RATa; NRAT; RAT; NRAT; ESD/CRT+E; ESD/CRT+EC; Home
1:- RATa; NRAT; RAT; NRAT; Home+EC
1:- RATa; NRAT; RAT; NRAT; Home
1:- RATa; NRAT; RAT; NRAT; NAIT; Carehome
1:- RATa; NRAT; RAT; NRAT; NAIT; ESD/CRT+C
1:- RATa; NRAT; RAT; NRAT; NAIT; Home+C
1:- RATa; NRAT; RAT; NRAT; NAIT; Non-SSNAPIn
1:- RATa; NRAT; RAT; NRAT; NRAT; Carehome+C
1:- RATa; NRAT; RAT; NRAT; NRAT; Non-SSNAPIn
1:- RATa; NRAT; RAT; NRAT; NRAT; Elsewhere
1:- RATa; NRAT; RAT; NRAT; RAT; NRAT; Carehome
1:- RATa; NRAT; RAT; NRAT; Non-SSNAPIn
1:- RATa; NRAT; RAT; NRAT; Elsewhere
1:- RATa; NRAT; RAT; Non-SSNAPESD/CRT
1:- RATa; NRAT; RAT; Non-SSNAPIn+C
1:- RATa; NRAT; RAT; Non-SSNAPIn
1:- RATa; RAT; ESD/CRT+E; ESD/CRT+C; Died
1:- RATa; RAT; ESD/CRT+E; ESD/CRT+C; ESD/CRT+C; Home
1:- RATa; RAT; ESD/CRT+E; ESD/CRT+C; Non-SSNAPESD/CRT
1:- RATa; RAT; ESD/CRT+EC; ESD/CRT+C; Home
1:- RATa; RAT; ESD/CRT+EC; ESD/CRT+EC; Home
1:- RATa; RAT; NAIT; ESD/CRT+C; Elsewhere
1:- RATa; RAT; NAIT; RAT; NAIT; ESD/CRT+E; Home
1:- RATa; RAT; NAIT; Non-SSNAPESD/CRT+EC
1:- RATa; RAT; NAIT; Non-SSNAPIn+C
1:- RATa; RAT; NAIT; Elsewhere+E
1:- RATa; RAT; NAIT; Elsewhere+EC
1:- RATa; RAT; NRAT; ESD/CRT+E; Died
1:- RATa; RAT; NRAT; ESD/CRT+E; Home
1:- RATa; RAT; NRAT; Home+EC
1:- RATa; RAT; NRAT; Home
1:- RATa; RAT; NRAT; NRAT; Home+EC
1:- RATa; RAT; NRAT; NRAT; Home
1:- RATa; RAT; NRAT; NRAT; NAIT; Home
1:- RATa; RAT; NRAT; RAT; Died
1:- RATa; RAT; NRAT; RAT; Home+C
1:- RATa; RAT; NRAT; RAT; Non-SSNAPIn+C
1:- RATa; RAT; NRAT; Non-SSNAPESD/CRT+E
1:- RATa; RAT; NRAT; Non-SSNAPIn
1:- RATa; RAT; NRAT; Elsewhere+EC
1:- RATa; RAT; RAT; Carehome+E
1:- RATa; RAT; RAT; ESD/CRT+C
1:- RATa; RAT; RAT; ESD/CRT+E
1:- RATa; RAT; RAT; ESD/CRT+E; Elsewhere
1:- RATa; RAT; RAT; ESD/CRT+EC
1:- RATa; RAT; RAT; ESD/CRT+EC; ESD/CRT+EC; Home

1:- RATa; RAT; RAT; ESD/CRT+EC; Home
1:- RATa; RAT; RAT; ESD/CRT+EC; Elsewhere
1:- RATa; RAT; RAT; NAIT; ESD/CRT+C
1:- RATa; RAT; RAT; NAIT; ESD/CRT+EC; Home
1:- RATa; RAT; RAT; NAIT; Home+C
1:- RATa; RAT; RAT; NAIT; Home
1:- RATa; RAT; RAT; NAIT; Non-SSNAPIn
1:- RATa; RAT; RAT; NRAT; ESD/CRT+C; Non-SSNAPESD/CRT
1:- RATa; RAT; RAT; NRAT; Home
1:- RATa; RAT; RAT; NRAT; Elsewhere+EC
1:- RATa; RAT; RAT; RAT; ESD/CRT+E; Elsewhere
1:- RATa; RAT; RAT; Elsewhere+C
1:- RATa; RAT; Non-SSNAPESD/CRT+EC
1:- RATa; RAT; Non-SSNAPIn+E
1:- RATa; RAT; Non-SSNAPIn+EC
1:- RATa; Non-SSNAP; ESD/CRT; Home
1:- RATc; ESD/CRT+C; ESD/CRT+C; Died
1:- RATc; ESD/CRT+C; ESD/CRT+EC
1:- RATc; ESD/CRT+C; ESD/CRT+EC; Home
1:- RATc; ESD/CRT+C; Non-SSNAPESD/CRT
1:- RATc; ESD/CRT+E; ESD/CRT+C; Died
1:- RATc; ESD/CRT+E; ESD/CRT+E
1:- RATc; ESD/CRT+E; ESD/CRT+E; Home
1:- RATc; ESD/CRT+E; ESD/CRT+EC; Carehome
1:- RATc; ESD/CRT+EC; ESD/CRT+C; Carehome
1:- RATc; Home+E; Home+E
1:- RATc; Home; Home
1:- RATc; NAIT; ESD/CRT+C; Non-SSNAPESD/CRT
1:- RATc; NAIT; ESD/CRT+E; Died
1:- RATc; NAIT; ESD/CRT+E; ESD/CRT+C
1:- RATc; NAIT; ESD/CRT+E; ESD/CRT+C; Home
1:- RATc; NAIT; ESD/CRT+EC; Died
1:- RATc; NAIT; NAIT; ESD/CRT+C
1:- RATc; NAIT; NAIT; ESD/CRT+C; Carehome
1:- RATc; NAIT; NRAT; Carehome+C
1:- RATc; NAIT; NRAT; Home+C
1:- RATc; NAIT; RAT; Carehome+C
1:- RATc; NAIT; RAT; ESD/CRT+EC; Home
1:- RATc; NAIT; RAT; Home
1:- RATc; NAIT; RAT; NAIT; Home
1:- RATc; NAIT; RAT; NAIT; Elsewhere
1:- RATc; NAIT; Non-SSNAPESD/CRT
1:- RATc; NAIT; Non-SSNAPIn+E
1:- RATc; NAIT; Non-SSNAPIn+EC
1:- RATc; NRAT; Carehome+E
1:- RATc; NRAT; ESD/CRT+E; ESD/CRT+C
1:- RATc; NRAT; Home+E
1:- RATc; NRAT; NAIT; Died
1:- RATc; NRAT; NAIT; ESD/CRT+C; Home
1:- RATc; NRAT; RAT; Home+EC
1:- RATc; NRAT; Non-SSNAPIn+E

1:- RATc; RAT; ESD/CRT+C; Carehome
1:- RATc; RAT; ESD/CRT+C; ESD/CRT+C; Home
1:- RATc; RAT; ESD/CRT+C; Elsewhere
1:- RATc; RAT; ESD/CRT+E; ESD/CRT+C
1:- RATc; RAT; ESD/CRT+EC; ESD/CRT+EC
1:- RATc; RAT; NAIT; Carehome+C
1:- RATc; RAT; NAIT; ESD/CRT+E; Carehome
1:- RATc; RAT; NAIT; ESD/CRT+EC; Non-SSNAPESD/CRT
1:- RATc; RAT; NAIT; Elsewhere
1:- RATc; RAT; RAT; Carehome+C
1:- RATc; RAT; RAT; ESD/CRT+C
1:- RATc; RAT; RAT; ESD/CRT+C; Home
1:- RATc; RAT; RAT; ESD/CRT+E; ESD/CRT+C; Home
1:- RATc; RAT; RAT; ESD/CRT+EC; Carehome
1:- RATc; RAT; RAT; Home+EC
1:- RATc; RAT; RAT; NAIT; Home+C
1:- RATc; RAT; RAT; NAIT; Home+EC
1:- RATc; RAT; RAT; NAIT; Non-SSNAPESD/CRT+C
1:- RATc; RAT; RAT; NAIT; Elsewhere
1:- RATc; RAT; RAT; RAT; ESD/CRT+E; ESD/CRT+C; Home
1:- RATc; RAT; RAT; Non-SSNAPESD/CRT+E
1:- RATc; RAT; Non-SSNAPESD/CRT+C
1:- RATc; RAT; Non-SSNAPESD/CRT+E
1:- RATc; RAT; Elsewhere+E
