# Supplementary material for: How do patients pass through stroke services? Identifying stroke care pathways using national audit data
Source: Clin Rehabil. 2020 Mar 6;34(5):698–709. doi: 10.1177/0269215520907654 (PMC7443957; doi:10.1177/0269215520907654)
Supplement: Supplementary_Data_Costs_by_pathway_and_severity – Supplemental material for How do patients pass through stroke services? Identifying stroke care pathways using national audit data [file Supplementary_Data_Costs_by_pathway_and_severity.pdf]

Supplementary Data. Costs of stroke care in each pathway, detailed by stroke severity .

	Stroke severity	No. of patients	% of independent patients at discharge (mRS <=2)	Average cost of inpatient care/ patient (£)*	Average cost of community based therapy/ patient (£)*	Total average cost/ patient (£)
Pathway 1	mild	7,498	58.00	2501.73	0.00	2501.73
Pathway 1	moderate	7978	27.95	4600.64	0.00	4600.64
Pathway 1	severe	2833	16.25	5184.26	0.00	5184.26
Pathway 1	very severe	3337	2.40	4231.75	0.00	4231.75
Pathway 1	Overall	21,646	26.15	4129.60	0.00	4129.60
Pathway 2	mild	43292	18.68	2949.71	0.00	2949.71
Pathway 2	moderate	86584	21.35	3371.10	0.00	3371.10
Pathway 2	severe	173168	24.40	3852.68	0.00	3852.68
Pathway 2	very severe	2863	2.46	5642.34	0.00	5642.34
Pathway 2	Overall	305907	16.72	3953.96	0.00	3953.96
Pathway 3	mild	7,895	60.06	2,502.88	331.32	2,834.20
Pathway 3	moderate	7,300	42.33	4,602.52	422.69	5,025.21
Pathway 3	severe	1,229	35.53	5,184.26	315.51	5,499.78
Pathway 3	very severe	884	12.68	4,233.34	267.56	4,500.90

Pathway 3	Overall	17,308	37.65	4,130.75	334.27	4,465.02
Pathway 4	mild	8,003	57.62	2,501.88	308.15	2,810.03
Pathway 4	moderate	6,908	40.94	4,601.93	295.46	4,897.40
Pathway 4	severe	1,307	17.60	5,185.07	289.74	5,474.81
Pathway 4	very severe	1,032	8.31	5,646.90	245.62	5,892.51
Pathway 4	overall	17,250	31.12	4,483.94	284.74	4,768.69
Pathway 5	mild	1425	34.75	5887.67	0.00	5887.67
Pathway 5	moderate	2859	17.69	9283.41	0.00	9283.41
Pathway 5	severe	1080	5.31	9127.28	0.00	9127.28
Pathway 5	v severe	1115	3.08	6761.50	0.00	6761.50
Pathway 5	overall	6479	15.21	7764.97	0.00	7764.97
Pathway 6	mild	262	30.83	7238.43	0.00	7238.43
Pathway 6	moderate	466	20.40	10474.94	0.00	10474.94
Pathway 6	severe	190	4.84	8612.15	0.00	8612.15
Pathway 6	very severe	149	7.06	7746.61	0.00	7746.61
Pathway 6	overall	1067	15.78	8518.03	0.00	8518.03

Pathway 7	mild	2626	43.51	6161.47	722.54	6884.02
Pathway 7	moderate	3922	29.61	9480.80	829.95	10310.75
Pathway 7	severe	1041	12.89	9955.35	535.37	10490.71
Pathway 7	very severe	821	8.52	6895.21	402.68	7297.89
Pathway 7	overall	8,410	23.63	8123.21	622.63	8745.84
Pathway 8	mild	501	38.98	7177.22	850.34	8027.56
Pathway 8	moderate	866	32.44	9988.87	651.38	10640.25
Pathway 8	severe	258	11.89	8281.75	674.76	8956.51
Pathway 8	very severe	185	12.57	7677.77	469.43	8147.20
Pathway 8	overall	1810	23.97	8281.40	661.48	8942.88
Pathway 9	mild	466	47.44	2981.30	368.64	3349.94
Pathway 9	moderate	461	24.28	5327.40	355.11	5682.51
Pathway 9	severe	117	7.03	3503.18	311.64	3814.82
Pathway 9	very severe	111	2.08	4460.85	1153.23	5614.08
Pathway 9	overall	1155	20.21	4068.18	547.16	4615.33
